# Determinants of coaches’ intentions to provide different recommendations on sports nutrition to their athletes

**DOI:** 10.1186/s12970-019-0311-x

**Published:** 2019-11-27

**Authors:** Raphaëlle Jacob, Steven Couture, Benoît Lamarche, Véronique Provencher, Éliane Morissette, Pierre Valois, Claude Goulet, Vicky Drapeau

**Affiliations:** 1 Institute of Nutrition and Functional Foods, Laval University, Quebec, G1V 0A6 Canada; 2 School of Nutrition, Laval University, Quebec, G1V 0A6 Canada; 3 Quebec Heart and Lung Institute Research Center, Quebec, G1V 4G5 Canada; 4 Centre de recherche interuniversitaire sur la formation et la profession enseignante (CRIFPE-Laval), Laval University, Quebec, G1V 0A6 Canada; 5 Department of Educational Fundamentals and Practices, Laval University, Quebec, G1V 0A6 Canada; 6 Department of Physical Education, Laval University, Québec, G1V 0A6 Canada

**Keywords:** Coaches, Adolescent athletes, Theory of planned behaviour, Sports nutrition

## Abstract

**Background:**

Coaches are considered as an important source of nutrition information by their athletes. However, their knowledge in this area is often insufficient for proper guidance and may lead to the dissemination of misinformation regarding sports nutrition. The aim of this study was to assess coaches’ intentions as well as psychosocial determinants underlying their intentions to provide sports nutrition recommendations to their high school athletes.

**Methods:**

Coaches (*n* = 47) completed a Web-based questionnaire based on the theory of planned behaviour, to assess their intentions to provide three different sports nutrition recommendations and their determinants. Multiple regression analyses were used to identify the variables that were most strongly related to the intentions.

**Results:**

Forty-six, 44.7 and 91.9% of coaches had the intention to recommend a higher consumption of foods rich in carbohydrates, foods rich in proteins and an increase in hydration to their athletes, respectively. Subjective norm was the only significant determinant of coaches’ intention to recommend a higher consumption of foods rich in carbohydrates (*R*^2^ = 53.7%, β = 0.73 ± 0.12, *P <* 0.0001). Subjective norm and perceived behavioural control were significantly associated with coaches’ intentions to recommend a higher consumption of foods rich in proteins (*R*^2^ = 25.9%, β = 0.50 ± 0.16, *P* = 0.002 and *R*^2^ = 17.2%, β = 0.39 ± 0.17, *P* = 0.01, respectively) and an increase in hydration (*R*^2^ = 26.8%, β = 0.38 ± 0.13, *P* = 0.001 and *R*^2^ = 46.3%, β = 0.58 ± 0.11, *P* < 0.0001, respectively).

**Conclusions:**

The results of this study suggest that subjective norm and perceived behavioural control represent important determinants of coaches’ intentions to provide recommendations on sports nutrition. These findings should be considered in future interventions aimed at facilitating proper general sports nutrition recommendations provided by coaches to their athletes.

## Background

Coaches have many responsibilities to promote the optimal development of their athletes and providing recommendations on sports nutrition certainly represent one important issue. Although family, friends, physicians, dietitians, the Internet, magazines and media represent important sources of information for high school athletes [1–7], most of them obtain their information regarding sports nutrition and dietary supplements mainly from their coaches [1–4, 6, 7]. Therefore, it is not surprising to see that many coaches provide general recommendations on sports nutrition to their athletes [8–10].

One important related issue is that most coaches do not have specific or formal training in sports nutrition, and their knowledge is often inadequate to appropriately guide their athletes on nutrition topics [8–12]. For instance, a study among French Canadian high school coaches showed that their nutrition knowledge is not optimal, yet, they reported providing advice on carbohydrates, proteins and hydration to their athletes [10]. Although a coach’s role is not to be an expert in sports nutrition, their close and daily interactions with their athletes justify the need to possess nutrition knowledge and skills to adequately guide their young athletes on general sports nutrition. Moreover, some athletes have access to a sport dietitian, but this support is not as easily accessible, and especially not on a regular basis, to most young athletes compared to high performance athletes. Therefore, there is a need to develop nutrition interventions for coaches based on a current evaluation of their recommendations on sports nutrition. These interventions are also justified by the suboptimal eating habits of adolescent athletes to meet requirements of daily training and ensure growth and health [13–18].

In order to develop an efficient intervention, it is essential to rely on a theoretical framework which allows a better understanding of the determinants of the targeted behaviour [19], i.e., general sports nutrition recommendations provided by coaches. The theory of planned behaviour (TPB) has proven its efficacy in terms of predicting human social behaviours [20] and was the theory most often used to assess the intentions and behaviours of healthcare professionals [21]. To our knowledge, no study has yet assessed the psychosocial determinants of sports nutrition recommendations provided by French Canadian high school coaches. This will provide valuable information for the development of a novel intervention for coaches based on their own beliefs and aimed at improving their nutrition knowledge and the recommendations they provide to their athletes.

In this context, the aim of this study was to extend our understanding of French Canadian high school coaches’ nutritional practices by 1) assessing coaches’ intentions to provide three different recommendations on sports nutrition to their athletes (i.e., a higher consumption of foods rich in carbohydrates, foods rich in proteins and an increase in hydration) and 2) identifying the determinants underlying their intentions to provide these recommendations. Based on a previous study which showed that protein and hydration were the most provided sports nutrition recommendations [10], it can be hypothesized that most coaches have the intention to provide these recommendations. At this time, it is premature to identify a hypothesis related to specific determinants of coaches’ intention to provide these recommendations since this has never been studied.

## Methods

### Participants and procedures

Participants were 47 coaches working for academic or extracurricular programs from five high schools in the Quebec City area, representing the same sample of high school coaches as Couture et al. (2015) [10]. School Offices were contacted to obtain consent and coaches were then recruited through emails and posters distributed in these high schools in 2011. To be included in the study, coaches had to work with athletes aged between 12 and 17 years at a competitive level (i.e., local to international competitive levels). The Research Ethics Committee of Laval University approved study procedures and written informed consent was obtained from all participants. After consent, a Web-link was sent to each participant in order to complete a Web-based questionnaire.

### Development of the web-based questionnaire

A Web-based questionnaire was developed in French based on the TPB specific guidelines for questionnaire development [22, 23], and on a previous related validated questionnaire [24]. The questionnaire assessed sociodemographic characteristics, intentions and determinants of coaches’ intentions to provide the three following sports nutrition recommendations to their athletes: 1) higher consumption of foods rich in carbohydrates to improve sport performance, 2) higher consumption of foods rich in proteins to enhance muscle gain, and 3) increase in hydration to improve sport performance. These topics were selected based on main sports nutrition guidelines [25] and on investigators’ professional experience. A list of the main sources of carbohydrates (i.e., grain products, fruit and vegetables and dairy products and alternatives) and proteins (i.e., meat, chicken, eggs, fish, nuts and seeds, legumes, and dairy products and alternatives) in foods as well as sources of hydration (i.e., water, fruit juices, sport drinks) was provided at the beginning of the questionnaire to ensure clarity of questions. The questionnaire also measured past behaviour towards these recommendations and coaches’ nutrition knowledge. Related results are presented elsewhere [10]. The questionnaire was piloted in four coaches to ensure understanding and adjustments were made when required.

### Theory of planned behaviour constructs

**Fig. 1:**
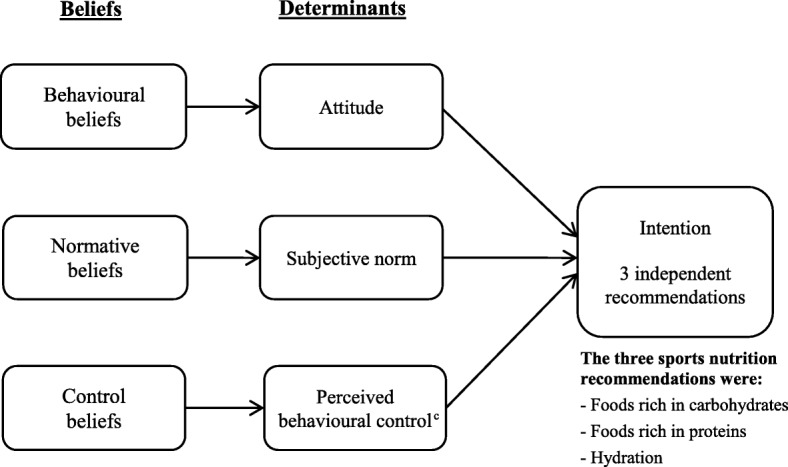
Theoretical framework used to identify the determinants of the intention of each sports nutrition recommendation

The TPB postulates that coaches’ intention to provide each of the three recommendations on sports nutrition to their athletes can be predicted by three psychosocial determinants, i.e., attitude, subjective norm and perceived behavioural control. Attitude refers to the perceived advantages and disadvantages towards a given behaviour [23]. Subjective norm is defined as people’s perception of what important referents think they should do [23]. Finally, perceived behavioural control is defined as the perceptions about the presence of factors that facilitate or impede the adoption of a given behaviour [23]. These determinants are based on behavioural, normative and control (i.e., barriers and facilitating factors) beliefs, respectively (Fig. 1).

#### Intention

Intention to provide sports nutrition recommendations within the next 3 months was assessed using one item for each of the three sports nutrition recommendations on a 6-point scale (totally disagree, 1, to totally agree, 6). For example, the following item was used: “In the next three months, I intend to recommend a higher consumption of foods rich in carbohydrates to my athletes to improve sport performance”.

#### Attitude and behavioural beliefs

Attitude was assessed as the mean of three items on a 6-point scale (ranging from 1 to 6). For example, participants were asked: “According to you, recommending a higher consumption of foods rich in carbohydrates to your athletes to improve sport performance in the next three months would be (...)”. Adjectives to qualify attitude were: useless/useful, bad/good, unacceptable/acceptable (Cronbach’s  α= 0.95, 0.94 and 0.89 for carbohydrates, proteins and hydration, respectively). Behavioural beliefs were assessed with four to nine items on a 4-point scale (not at all, 1, to enormously, 4). For instance, coaches were asked: “In the next three months, if you recommend an increase in the consumption of foods rich in carbohydrates to your athletes, do you think it would contribute to: (e.g., improve their athletic performance)” (Cronbach’s α  = 0.74, 0.81 and 0.62 for carbohydrates, proteins and hydration, respectively). Of note, behavioural beliefs towards hydration have not been used in the analysis because attitude was not a significant determinant of coaches’ intention to recommend an increase in hydration. Moreover, knowing that it is possible to have and hold contradictory beliefs, both positive and negative, about a given behaviour, internal consistency coefficient, such as Cronbach’s α, should be interpreted with caution in this context [26].

#### Subjective norm and normative beliefs

Subjective norm was assessed as the mean of three items on a 6-point scale (strongly disagree or disapprove, 1, to strongly agree or approve, 6). For example, the following statement was used: “People that are important to you think you should recommend a higher consumption of foods rich in carbohydrates to your athletes to improve sport performance within the next three months.” (Cronbach’s α= 0.86, 0.92 and 0.61 for carbohydrates, proteins and hydration, respectively). Normative beliefs were assessed with seven items on a 4-point scale (strongly unfavourable, 1, to strongly favourable, 4) with a non-applicable option. For example, participants were asked: “To what extent would the following people (e.g., your athletes) be favourable or unfavourable to the idea that you steadily recommend a higher consumption of foods rich in carbohydrates to your athletes in the next three months to improve sport performance?” (Cronbach’s  α= 0.98, 0.99, 0.99 for carbohydrates, proteins and hydration, respectively).

#### Perceived Behavioural control and control beliefs

Perceived behavioural control was assessed as the mean of three items on a 6-point scale (strongly disagree, 1, to strongly agree, 6). For example, participants were asked: “If you wanted to, you could easily recommend a higher consumption of foods rich in carbohydrates to your athletes within the next three months to increase sport performance.” (Cronbach’s α= 0.84, 0.86 and 0.75 for carbohydrates, proteins and hydration, respectively). Up to seven barriers and facilitating factors related to each recommendation were used to measure control beliefs. For example, participants were asked to rate the following question on a 6-point scale (strongly unlikely, 1, to strongly likely, 6): “Do you think that it is unlikely or likely that the following factors will encourage you (or discourage you, for barriers, e.g., lack of knowledge) to recommend a higher consumption of foods rich in carbohydrates to your athletes, in the next three months, to improve sport performance?” (Cronbach’s  α= 0.95, 0.95 and 0.90 for carbohydrates, proteins and hydration’ facilitating factors, respectively; and 0.91, 0.86 and 0.77 for carbohydrates, proteins and hydration’ barriers, respectively.).

### Statistical analysis

Participant characteristics are presented as frequency. To assess the prevalence of intentions, arbitrary groups for intention to provide each recommendation were defined as follows: having no intention (score 1 to 3) or having intention (score 4 to 6). A continuous score (1 to 6) was used to identify which psychosocial determinant(s) predicted the intention. Associations among 1) TPB determinants and intention and 2) beliefs related to each determinant were investigated using Pearson correlations. Stepwise multiple linear regression analyses were also used to identify the determinants of intention to provide each recommendation. Due to multicolinearity, ridge regressions were performed to identify the main beliefs associated with significant determinant(s) of each of the three intention measures; these significant determinants were identified in the previous step (Fig. 1). In ridge regression analyses, the non-applicable option related to normative beliefs was scored as the mean of participant’s answers to other normative beliefs items for each behaviour. In correlation analyses, the participant was not included in the analysis if the non-applicable option was answered for the related belief. Multiple regression and ridge regression analyses are presented as standardized β ± standard error (SE) and standardized β, respectively. To consider the heterogeneity of sports that could impact coaches’ intention to provide sports nutrition recommendations and their determinants, analyses were also performed according to two main sport groups, i.e., “leanness” where coaches were involved in aesthetic or endurance sports (e.g., gymnastics, athletics) and “non-leanness” where coaches were working mainly in team sports [27]. Because of the small sample size, the analyses according to sport groups should be considered as exploratory. Moreover, the analyses related to beliefs associated with determinants were not performed separately in the two sport groups due to a lack of power given the higher number of variables in each model. The retrospective sample size calculation indicated that 36 participants were required for multiple regressions, using a power of 80%, an alpha level of 0.05 and an effect size of 0.35, which is considered as a large effect size [28]. Sport group differences in participant characteristics and their intention were investigated using chi-squared analyses. Statistical analysis was performed using SAS version 9.4 (SAS Institute Inc., Cary, NC, USA) and R Core Team version 3.5.1 (The R Foundation for Statistical Computing, Vienna, Austria) and differences were considered significant at *P* < 0.05.

## Results

### Participant characteristics

Forty-seven coaches were recruited and completed the study. Twenty-nine and 18 coaches were involved in nonleanness and leanness sports, respectively (Table 1). Slightly over half were male (55.3%) with a mean age of 29.4 ± 9.5 years, ranging from 17 to 55 years. Their coaching experience ranged from 1 to 30 years, with an average of 9.4 ± 7.3 years. There was no difference in coaches’ age, sex, number of years of experience in coaching, education level and National Coaching Certification Program (NCCP) level in coaches involved in nonleanness vs. leaneness sports (data not shown, *P* > 0.05). However, coaches in leanness sports were more likely to coach national or international level athletes and female athletes than nonleanness coaches (data not shown, *P* = 0.002 and *P* < 0.0001, respectively).

**Table 1:** Participant characteristics

Characteristics	Frequency, *n* (%)
Sex	
Men	26 (55.3)
Women	21 (44.7)
Age	
< 30 years	21(56.8)
≥ 30 years	16 (43.2)
Experience in coaching	
≤ 10 years	27 (57.5)
> 10 years	20 (42.6)
Education level	
University	24 (51.1)
College or high school	23 (48.9)
NCCP level	
None	13 (27.7)
Levels 1–2	21 (44.7)
Levels 3–4	13 (27.7)
Type of sport^a^	
Nonleanness	29 (61.7)
Leanness	18 (38.3)
Coaching level	
Regional or provincial	17 (38.6)
National or international	27 (61.4)
Sex of athletes coached	
Female	16 (34.0)
Male	17 (36.2)
Mixed	14 (29.8)

NCCP: National Coaches Certification Program

^a^Nonleanness sports: football (*n* = 16), basketball (*n* = 8), badminton (*n* = 1), soccer (*n* = 2), tennis (*n* = 1), and alpine skiing (*n* = 1). Leanness sports: track and field (*n* = 1) cheerleading (*n* = 9), gymnastics (*n* = 2), synchronized swimming (*n* = 3), diving (*n* = 2), cross-country skiing (*n* = 1)

### Intention

**Fig. 2:**
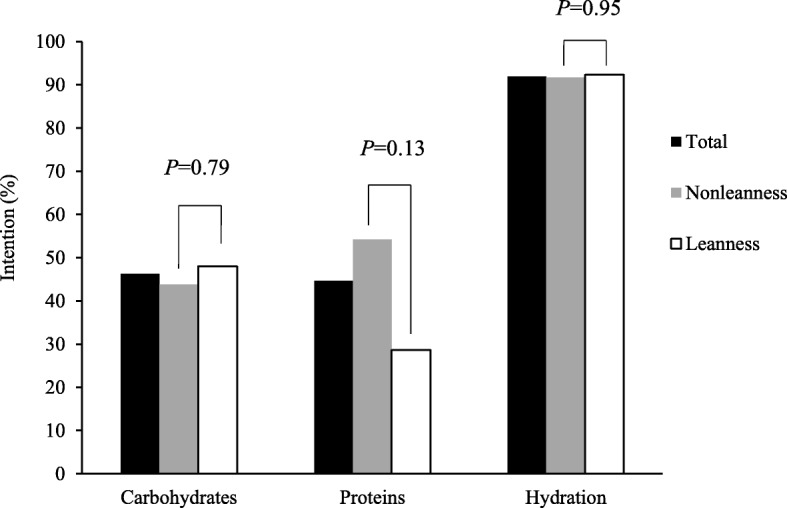
Prevalence of high school coaches having the intention to recommend a higher consumption of foods rich in carbohydrates, a higher consumption of foods rich in proteins and an increase in hydration to their athletes within the next 3 months. Intention to provide each recommendation was measured using arbitrary categories based on a 6-point scale defined as follows: Having no intention: score = 1 to 3; Having intention: score = 4 to 6. Total represents the whole sample of coaches having answered the question related to the intention to provide each of the three sports nutrition recommendations. Carbohydrates: Total *n* = 41; Nonleanness *n* = 25; Leanness *n* = 16. Proteins: Total *n* = 38; Nonleanness *n* = 24; Leanness *n* = 14; Hydration: Total *n* = 37; Nonleanness *n* = 24; Leanness *n* = 13

Forty-six (46.3) and 44.7% of high school coaches had the intention to recommend a higher consumption of foods rich in carbohydrates and a higher consumption of foods rich in proteins, respectively (Fig. 2). A high proportion of coaches (91.9%) had the intention to recommend an increase in hydration. No significant differences were observed between nonleanness and leanness sports in the prevalence of coaches having the intention to recommend a higher consumption of carbohydrates (43.8%, vs. 48.0%, respectively, *P* = 0.79) and an increase in hydration (91.7% vs. 92.3%, respectively, *P* = 0.95). However, a higher number of nonleanness coaches seemed to have the intention to recommend a higher consumption of foods rich in proteins than leanness coaches, although the difference did not reach significance (54.2% vs. 28.6%, respectively, *P* = 0.13). No differences were observed in the proportion of coaches having the intention to recommend each of the three different sports nutrition practices depending on characteristics presented in Table 1, namely, sex and age of coaches, years of experience in coaching, education level, National Coaching Certification Program (NCCP) level, coaching level and sex of athletes (data not shown, *P >* 0.05).

### Psychosocial determinants

#### Associations between intention and its determinants

**Fig. 3:**
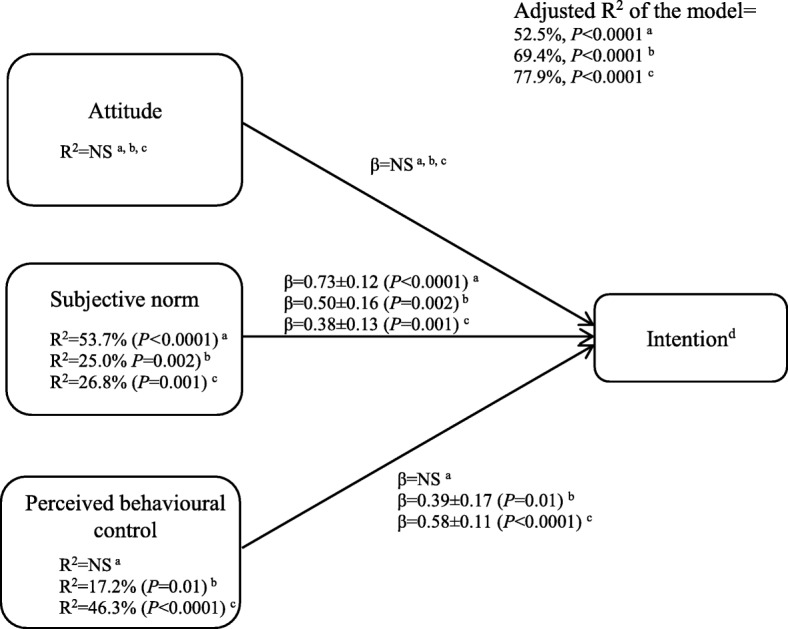
Multiple regressions of the determinants of coaches’ intention to recommend a higher consumption of foods rich in carbohydrates, a higher consumption of foods rich in proteins and an increase in hydration to their athletes within the next 3 months. NS, not significant at *P*<0.05. ^a^ Determinants of coaches’ intention to recommend a higher consumption of foods rich in carbohydrates (*n* = 40). ^b^ Determinants of coaches’ intention to recommend a higher consumption of foods rich in proteins (*n* = 37). ^c^ Determinants of coaches’ intention to recommend an increase in hydration (*n* = 37). ^d^ Intention to recommend a higher consumption of foods rich in carbohydrates, or a higher consumption of foods rich in proteins, or an increase in hydration, depending on the model (i.e., a, b, c)

All determinants showed a significant positive correlation with coaches’ intention to recommend 1) a higher consumption of foods rich in carbohydrates (*r* = 0.58 to 0.73, *P* < 0.0001), 2) a higher consumption of foods rich in proteins (*r* = 0.70 to 0.81, *P* < 0.0001), and 3) an increase in hydration (*r* = 0.78 to 0.85, *P* < 0.0001) to their athletes. Multiple regression analyses showed that subjective norm was the only significant determinant of coaches’ intention to recommend a higher consumption of foods rich in carbohydrates (Fig. 3, β = 0.73 ± 0.12, *P <* 0.0001), explaining 53.7% of its variance. Subjective norm and perceived behavioural control were singled out as significant determinants of coaches’ intention to recommend a higher consumption of foods rich in proteins (Fig. 3, *R*^2^ = 25.0%, β = 0.50 ± 0.16, *P* = 0.002 and *R*^2^ = 17.2%, β = 0.39 ± 0.17, *P* = 0.01, respectively) and an increase in hydration (Fig. 3, *R*^2^ = 26.8%, β = 0.38 ± 0.13, *P* = 0.001 and *R*^2^ = 46.3%, β = 0.58 ± 0.11, *P* < 0.0001, respectively).

Analyses conducted separately among the two groups of sports yielded the same results as for the whole group regarding the determinant of coaches’ intention to recommend a higher consumption of foods rich in carbohydrates. Indeed, subjective norm was the only significant determinant of coaches’ intention to recommend a higher consumption of foods rich in carbohydrates in both nonleanness and leanness groups (*R*^2^ = 40.9%, β = 0.64 ± 0.21, *P* = 0.0006 and *R*^2^ = 73.2%, β = 0.86 ± 0.14, *P* < 0.0001, respectively). Regarding the intention to recommend a higher consumption of foods rich in proteins, subjective norm was the only significant determinant in nonleanness coaches (*R*^2^ = 55.6%, β = 0.75 ± 0.17, *P <* 0.0001) whereas intention was explained by perceived behavioural control in leanness coaches (*R*^2^ = 74.7%, β = 0.75 ± 0.13, *P =* 0.0001). Intention to recommend an increase in hydration was associated with perceived behavioural control and subjective norm in nonleanness coaches (*R*^2^ = 57.2%, β = 0.65 ± 0.13, *P <* 0.0001 and *R*^2^ = 27.2%, β = 0.34 ± 0.18, *P* = 0.01, respectively) and with subjective norm in leanness coaches (*R*^2^ = 37.2%, β = 0.56 ± 0.24, *P* = 0.04). Moreover, there were no group of sport differences in the mean value of each TPB determinants for the recommendation of carbohydrates and hydration (data not shown, *P* > 0.05). However, all TPB determinants (mean value) related to the recommendation of proteins were significantly higher among nonleanness coaches (data not shown, *P* < 0.05).

#### Specific beliefs related to each significant determinant of intention

Correlation analyses showed that all referents representing normative beliefs were significantly associated with subjective norm towards the recommendations of carbohydrates, proteins and hydration (Table 2). In ridge regression analysis, the two most important determinants of subjective norm towards the recommendation of carbohydrates were coaches’ beliefs about what parents of their athletes (β = 0.51) and colleague coaches (β = 0.40) think they should do. Similar results were observed for the recommendations of foods rich in protein, with coaches’ belief about what parents of their athletes think they should do being the most important referent towards subjective norm (β = 0.70) followed by their athletes (β = 0.49). For the recommendation of hydration, none of the referents remained in the model (Table 2).

**Table 2:** Associations among normative beliefs and subjective norm towards the recommendations to increase the consumption of foods rich in carbohydrates and proteins and to increase hydration

Normative belief	Carbohydrates				Proteins				Hydration		
	*r* ^1^	*p* ^*2*^	β ^3^		*r* ^1^	*p* ^*2*^	β ^3^		*r* ^1^	*p* ^*2*^	β ^3^
Team/club physician ^4^	0.67	< 0.0001	–		0.58	0.002	–		0.84	< 0.0001	–
Colleagues coaches of the team/club ^5^	0.72	< 0.0001	0.40		0.68	< 0.0001	–		0.52	0.001	–
Parents of athletes ^6^	0.73	< 0.0001	0.51		0.72	< 0.0001	0.70		0.52	0.001	–
Team/club dietitian ^7^	0.74	< 0.0001	0.07		0.59	0.003	–		0.88	< 0.0001	–
Athletes ^8^	0.66	< 0.0001	0.03		0.73	< 0.0001	0.49		0.53	0.0009	–
Leaders of the provincial sport federation ^9^	0.64	< 0.0001	–		0.59	0.0007	–		0.57	0.002	–
Leaders of the team/club ^10^	0.63	< 0.0001	–		0.64	< 0.0001	–		0.56	0.001	–

^1^Values are Pearson correlation coefficients

^2^*P* values related to Pearson correlations

^3^β coefficient from ridge regression

^4^Pearson correlation *n* = 26 to 29; ^5^ Pearson correlation *n* = 36 to 40; ^6^ Pearson correlation *n* = 37 to 39; ^7^ Pearson correlation *n* = 22 to 28; ^8^ Pearson correlation *n* = 35 to 39; ^9^ Pearson correlation *n* = 28 to 33; ^10^ Pearson correlation *n* = 32 to 38. Ridge regression: Carbohydrates *n* = 40; Protein *n* = 37; Hydration *n* = 37

None of the barriers were associated with perceived behavioural control towards the recommendation of food rich in proteins in correlation analyses or ridge regression (data not shown), but all facilitating factors were associated with perceived behavioural control regarding this recommendation (Table 3). In ridge regression analysis, the beliefs “if other coaches recommend the increase of food rich in proteins to their athletes” (β = 0.54), “if it would make your athletes better” (β = 0.46), and “the information sessions held by professionals in the field” (β = 0.44) were identified as the most important facilitating factors associated with perceived behavioural control towards protein recommendation. None of the barriers were associated with perceived behavioural control regarding the recommendation of hydration in Pearson correlations or ridge regression (data not shown). Similarly, the facilitating factors were not significantly correlated with perceived behavioural control towards the recommendation of hydration and ridge regression showed a trivial effect of each facilitating factor (β = 0.01 to 0.02).

**Table 3:** Associations among facilitating factors and perceived behavioural control towards the recommendation to increase the consumption of foods rich in proteins

Facilitating factors	*r* ^1^	*p* ^*2*^	β ^3^
If a colleague coach advises you to recommend the increase of foods rich in proteins to your athletes	0.42	0.01	−0.10
If other coaches recommend the increase of foods rich in proteins to their athletes	0.53	0.0007	0.54
If it would make your athletes better	0.54	0.0006	0.46
If it would ensure your team to qualify for the provincial/national championship	0.44	0.006	−0.09
The information sessions held by professionals in the field	0.51	0.001	0.44
The information found in magazines, ads, journals and the Internet	0.34	0.04	−0.27

^1^Values are Pearson correlation coefficients (*n* = 36 to 37)

^2^*P* values related to Pearson correlations

^3^β coefficient from ridge regression (*n* = 36)

## Discussion

This study aimed to assess high school coaches’ intention to provide three different sports nutrition recommendations to their athletes as well as to identify the determinants and their underlying beliefs of coaches’ intention towards these recommendations. Results showed that a higher number of coaches had the intention to recommend hydration than the consumption of foods rich in carbohydrates or rich in proteins to their athletes. Subjective norm and perceived behavioural control were identified as the main determinants of these intentions. To our knowledge, this study is the first to investigate coaches’ intention to provide recommendations on sports nutrition and their related determinants and beliefs. These findings are important since they represent theoretical foundations of future interventions aimed at facilitating proper general sports nutrition recommendations provided by high school coaches to their young athletes (e.g., [29]).

In this sample of high school coaches, 46.3% reported having the intention to recommend a higher consumption of foods rich in carbohydrates to their athletes in the next 3 months, and the prevalence was not different depending on leanness or nonleanness sports. This result cannot be compared to other studies, since, to our knowledge, this study is the first to assess the intention regarding sports nutrition recommendations provided by coaches. It is, however, consistent with the prevalence of coaches (i.e., 47.6%) having reported that they had recommended the consumption of foods rich in carbohydrates during the last 12 months to their athletes (i.e., past behaviour) from the same sample of coaches [10]. Considering that carbohydrate is the main source of energy for most exercise, it is important that athletes consumed this nutrient in a sufficient amount to support sport performance. As reported in the study of Lun et al. (2009), high performance Canadian athletes had daily carbohydrate intake of 5.1 ± 1.8 g/kg of body weight [30], indicating that most athletes do not meet carbohydrate recommendations for moderate or high endurance exercise program (5–12 g/kg/d) according to guidelines [31]. Similar results have been observed in adolescent athletes [15, 16, 32, 33]. For example, 41% of club level male tennis players from Brazil (age 14–18 years) and 52% of junior elite Canadian female soccer athletes (age 15.7 ± 0.7 years) reported carbohydrate intake below the recommended values [16, 33]. Insufficient carbohydrates during training have also been observed in skill (e.g., tennis) and team-sport adolescent athletes, as less than 30% consumed 30 to 60 g of carbohydrates per hour during training and competition [13]. Considering that a high proportion of athletes do not consume enough carbohydrates daily, and that coaches represent an important source of nutrition information and influence, the result suggests that the prevalence of coaches having the intention to recommend a higher consumption of foods rich in carbohydrates should be higher.

The prevalence of coaches having the intention to recommend a higher consumption of foods rich in proteins to their athlete (i.e., 44.7%) is similar to the one related to carbohydrates, but lower than the number of coaches having reported they had provided this recommendation in the last 12 months (i.e., 97.5%) [10]. In contrast to carbohydrate intakes, Lun et al. (2009) reported that Canadian athletes had a mean daily protein intake of 1.8 ± 0.6 g/kg of body weight [30], meaning that most of them reach protein recommendations (i.e., 1.2–2.0 g/kg/d) [31]. Protein intake above the minimum recommended value of 1.2 g/kg/d was also observed in most adolescent athletes [15, 16, 32, 33]. Therefore, the prevalence of coaches having the intention to recommend a higher consumption of foods rich in proteins could be considered as reasonable.

Subjective norm was identified as the main correlate of coaches’ intentions to recommend a higher consumption of foods rich in carbohydrates and foods rich in proteins to their adolescent athletes. These results suggest that perceived social pressure towards carbohydrate and protein recommendations has a stronger impact than the perceived advantages and disadvantages, or the perceived barriers and facilitating factors on the intention to adhere to these behaviours. Indeed, carbohydrate and protein recommendations before, during and after training or competition are more complex than hydration recommendations, and require a minimum of knowledge and abilities regarding general sports nutrition. Such constraints may explain why coaches are more subject to external influence. While all referents representing normative beliefs were significantly associated with subjective norm in correlation analyses, parents of athletes seemed to be an important referent of coaches’ intentions to recommend a higher consumption of foods rich in carbohydrates and foods rich in proteins to their athletes. It can be speculated that because parents of athletes aged 12–17 years are mostly responsible for food purchase and preparation [34] and they also represent an important source of nutrition information for athletes [6, 11], coaches rely on parents’ opinions for these behaviours. Future interventions should focus in educating coaches and developing their skills related to general sports nutrition so they could be more influenced by evidenced-based sources of nutrition information for these behaviours.

The high prevalence (i.e., 91.9%) of coaches having the intention to recommend an increase in hydration to improve performance seems appropriate since adequate fluid intake before, during and after exercise is important for health and optimal performance, though an inter-individual variability is now recognized on the impact of dehydration on sport performance [31]. Moreover, studies among adolescent athletes generally showed that they have suboptimal hydration practices [17, 35, 36]. This high prevalence of intention combined with all coaches having reported they had recommanded hydration to their athletes in the last 12 months [10] suggests that this recommendation is more common and less ambiguous. This may explain why perceived behavioural control was the most important determinant of coaches’ intention to recommend hydration. When a referent group performs a behaviour, which is considered as the descriptive norm, it may indirectly influence perceived behavioural control over this behaviour [23]. Because water is usually free and easily accessible, coaches might perceive few barriers to provide this recommendation.

Finally, the results of this study highlight group of sport differences in coaches’ intention to provide some specific sports nutrition recommendations, i.e., foods rich in proteins. Group of sport differences were also observed for determinants of coaches’ intention to recommend the consumption of foods rich in proteins and hydration. These differences may be explained by cultural aspects and physical demands of each type of sports.

### Strengths and limits

To our knowledge, this study is the first to assess coaches’ intentions to provide different sports nutrition recommendations to their athletes and the determinants of their intentions. In addition to the results on nutrition knowledge and nutritional practices provided by French Canadian high school coaches presented in Couture et al. 2015 [10], this study yields a broad understanding of coaches' sports nutrition practices representing key information for the development of training regarding general sports nutrition. Questions measuring behavioural, normative and control beliefs were based on the literature and on group discussions among researchers. Moreover, very few theoretical frameworks provide specific guidelines for questionnaire development, as does the TPB, which ensures the quality of the recorded data [22]. In future studies, focus groups could be used to identify modal behavioural, normative and control beliefs in a sample of the study population to ensure that all coaches’ beliefs are considered. The small number of coaches and the high proportion of football coaches also limit the generalization of results to all sports. The sample size was also a constraint for the consideration of specific determinants and beliefs related to the intention to recommend the three different sports nutrition practices in the two groups of sports. Specific sport cultural aspects also need to be considered, such as the use of protein supplements among young football players [37], which can influence coaches’ intention to recommend the sports nutrition practices assessed in the present study. Moreover, due to time and financial constraints, it was not possible to assess the actual sports nutrition recommendations provided by coaches, but the past behaviour was nonetheless assessed and presented elsewhere [10]. Although intention is viewed as the most proximal determinant of a behaviour, one cannot assume that coaches actually provide the recommendations assessed in this study, as a meta-analysis of meta-analyses showed that the intention account for an important part (i.e., 28%), but not all of the variance of a behaviour [38]. In this context, future studies should be performed with a larger cohort from a greater variety of sports and should assess the actual behaviour, i.e., recommendations on sports nutrition provided to their athletes, using tools such as a logbook or a diary.

## Conclusion

Coaches represent an important source of nutrition information by their athletes. The results of this study suggest that a greater number of high school coaches intend to recommend hydration than consuming carbohydrates or proteins to support sport performance. Subjective norm and perceived behavioural control were identified as key determinants of their intention to provide these sports nutrition recommendations. These findings should be considered in future educative interventions aimed at facilitating proper general sports nutrition recommendations provided by coaches to their young athletes. These interventions are highly needed considering that adolescent athletes have particular dietary needs and non-optimal dietary intakes, that coaches are an important source of influence and that sport dietitians are not as easily and regularly accessible at this sport level. We have recently shown that an intervention based on the determinants of coaches’ intentions to provide different recommendations on sports nutrition identified in the present study was effective in improving the recommendations provided by high school coaches and their nutrition knowledge [29].

## Data Availability

The datasets analysed during the current study are available from the corresponding author on reasonable request.
